# Robust neuroprotective effects of 2-((2-oxopropanoyl)oxy)-4-(trifluoromethyl)benzoic acid (OPTBA), a HTB/pyruvate ester, in the postischemic rat brain

**DOI:** 10.1038/srep31843

**Published:** 2016-08-22

**Authors:** Seung-Woo Kim, Hye-Kyung Lee, Il-Doo Kim, Hahnbie Lee, Lidan Luo, Ju-Young Park, Sung-Hwa Yoon, Ja-Kyeong Lee

**Affiliations:** 1 Department of Biomedical Sciences, Inha University School of Medicine, Inchon, Republic of Korea; 2 Medical Research Center, Inha University School of Medicine, Inchon, Republic of Korea; 3 Department of Anatomy, Inha University School of Medicine, Inchon, Republic of Korea; 4 Department of Molecular Science and Technology, Ajou University, Suwon, Republic of Korea

## Abstract

Postischemic brain damage in stroke is proceded with complicated pathological events, and so multimodal drug treatments may offer better therapeutic means for improving clinical outcomes. Here, we report robust neuroprotective effects of a novel compound, 2-((2-oxopropanoyl)oxy)-4-(trifluoromethyl)benzoic acid (OPTBA), a 2-hydroxy-4-trifluoromethyl benzoic acid (HTB, a metabolite of triflusal)-pyruvate ester. Intravenous administration of OPTBA (5 mg/kg) 3 or 6 h after middle cerebral artery occlusion (MCAO) in Sprague-Dawley rats reduced infarct volumes to 38.5 ± 11.4% and 46.5 ± 15.3%, respectively, of that of MCAO controls, and ameliorated motor impairment and neurological deficits. Importantly, neuroprotective effects of OPTBA were far greater than those afforded by combined treatment of HTB and pyruvate. Furthermore, OPTBA suppressed microglial activation and proinflammatory cytokine inductions more effectively than HTB/pyruvate co-treatment in the postischemic brain and LPS-treated cortical slice cultures and also attenuated NMDA-induced neuronal death in hippocampal slice cultures. LC-MS analysis demonstrated that OPTBA was hydrolyzed to HTB and pyruvate with a t_1/2_ of 38.6 min in blood and 7.2 and 2.4 h in cortex and striatum, respectively, and HTB was maintained for more than 24 h both in blood and brain tissue. Together these results indicate OPTBA acts directly and via its hydrolysis products, thus acting as a multimodal neuroprotectant in the postischemic brain.

In the postischemic brain, neuronal cell damage and subsequent neurological dysfunction are caused by complicated pathological events occurring in a spatiotemporally-regulated manner. Excitotoxicity and Zn^2+^ toxicity cause massive neuronal cell damages in the ischemic core during the acute phase^1^ and this is followed by inflammation and apoptosis within a few hours to days that exacerbate brain injury^2^. It is for this reason that combinatorial or multimodal drug treatments are believed to be most effective for stroke treatment. In this respect, it has been reported combination treatment with edaravone and borneol confers synergistic neuroprotective effects in the postischemic brain via anti-oxidative and anti-inflammatory mechanisms, respectively^3^. In addition, co-treatment with recombinant tissue plasminogen activator (rtPA) and minocycline (a PARP-1 inhibitor) was found to enhance protective effects by suppressing inflammation, infarction formation, brain swelling, and hemorrhage in focal embolic stroke^4^. In a previous study, we also reported combination treatment with ethyl pyruvate and aspirin acted synergistically to afford neuroprotection in the postischemic brain via the differential modulation of NF-κB signaling^5^. Subsequently, we introduced a multimodal neuroprotectant, OBA-09, a salicylic acid/pyruvate ester, which conferred robust neuroprotective effects in the postischemic brain by reducing ROS generation and suppressing excitotoxicity and Zn^2+^ toxicity^6^.

Triflusal (2-acetoxy-4-trifluoromethylbenzoic acid) is a 4-fluoromethyl derivative of aspirin and an anti-platelet drug that functions by directly inhibiting cyclooxygenase-2 (COX-2) and indirectly inhibiting NF-κB^7^. Like aspirin, which is an anti-thrombotic known to function at the molecular level as a COX-1/COX-2 inhibitor^8^, long-term treatment with triflusal was found to be effective for the prevention of secondary ischemic stroke, but safer than aspirin because of its lower hemorrhagic risk^9^. In addition to its protective effects in the ischemic brain, a study in a transgenic mouse model of Alzheimer’s disease showed that chronic treatment with triflusal reduced dense-cored plaque load and proinflammatory cytokine levels and rescued cognitive deficits^10^. It has also been reported triflusal inhibited COX-2 expression and PGE2 production in a rat carrageenan-induced air pouch model^11^ and that it reduced pro-inflammatory mediators, such as, iNOS, COX-2, and TNF-α after N-methyl-D-aspartate (NMDA)-induced postnatal excitotoxic damage^12^. Interestingly, its metabolite HTB was also found to suppress NF-κB activation in LPS-treated peripheral blood mononuclear cells (PBMC)^11^. In addition, recently, we reported that anti-inflammatory effects and overall neuroprotective potency of HTB in the postischemic brain are greater than those of triflusal (Kim *et al*., submitted).

Pyruvate (CH_3_COCOO^−^), the anionic form of the simplest alpha-keto acid, is a product of glycolysis, a substrate for the tricarboxylic acid (TCA) cycle, and is known to normalize NAD levels in Zn^2+^-treated neurons^13^. By acting as a fuel substrate to protect cells from ischemic injury, pyruvate prevents poly (ADP-ribose) polymerase 1 (PARP-1)-mediated cell death by inhibiting the activations of caspase and restoring intracellular ATP levels^14^. Yi *et al*.^15^ found systemic pyruvate suppressed infarct formation and improved motor deficits in an animal model of focal cerebral ischemia and Wang *et al*.^16^ reported pyruvate significantly reduced neutrophil infiltration and microglia activation and suppressed NF-κB activation in postischemic- and in LPS-administered rat brains.

In the present study, we synthesized a novel hybrid molecule, a simple ester of HTB and pyruvate, and examined its neuroprotective effects in a rat model of middle cerebral artery occlusion (MCAO). We examined hydrolysis kinetics of HTB in serum and brain tissue using LC-MS and also investigated molecular mechanisms underlying the neuroprotective effects, in particular, its anti-inflammatory and anti-excitotoxic effects, using hippocampal and cortical slice cultures (Fig. S1).

## Results

### LC-MS revealed slow hydrolysis of OPTBA and excellent stability of HTB in blood and brain tissue

**Figure 1:**
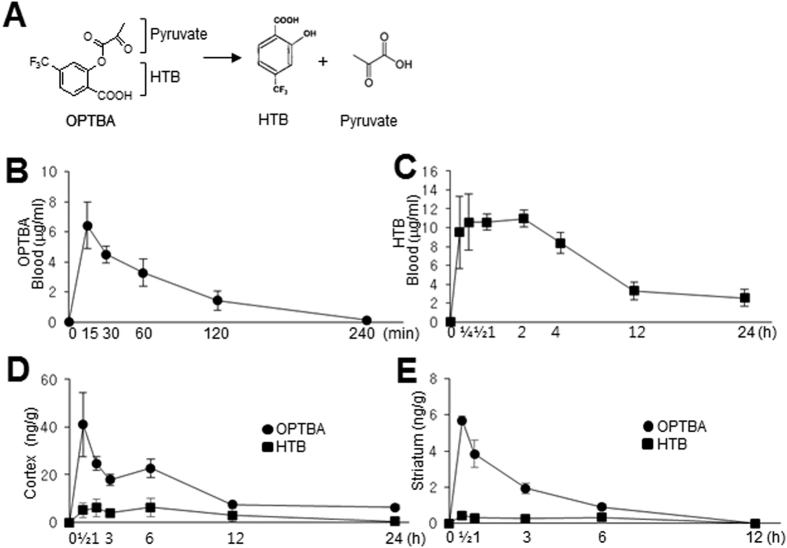
Kinetics of OPTBA hydrolysis and HTB release *in vivo*. (**A**) Structures of OPTBA and of its metabolic products, HTB and pyruvate. (**B**–**D**) OPTBA (5 mg/kg, i.v.) was injected intravenously into treatment-naive Sprague-Dawley rats and temporal concentration profiles of OPTBA and HTB in blood (**B**,**C**) and in brain tissue (cerebral cortex and striatum) (**D**,**E**) were monitored by LC/ESI-MS. Results are presented as means ± SEMs (n = 3).

In an effort to produce a multimodal neuroprotective drug, in particular, for treating cerebral ischemia, we synthesized a novel hybrid molecule named OPTBA, 2-((2-oxopropanoyl)oxy)-4-(trifluoromethyl)benzoic acid, an ester of HTB (a 2-hydroxy-4-trifluoromethyl benzoic acid) and pyruvate (Fig. 1A). To investigate the kinetics of HTB and pyruvate release from OPTBA, 5 mg/kg of OPTBA was administered intravenously (i.v.) to a treatment-naive Sprague-Dawley rat and LC/ESI-MS analysis was performed on serum or brain tissue (cerebral cortex and striatum) at 15, 30, 60, 120, and 240 min, and 12 and 24 h after OPTBA treatment. It was found that OPTBA was hydrolyzed with a t_1/2_ of 38.6 min in blood and of 7.2 h and 2.4 h in cortex and striatum, respectively (Fig. 1B–E). HTB surge was detected immediately after the administration and continued to be detected until 24 h in both blood and brain parenchyma (Fig. 1B–E).

### OPTBA suppressed infarct formation in the postischemic brain with an extended therapeutic window

**Figure 2:**
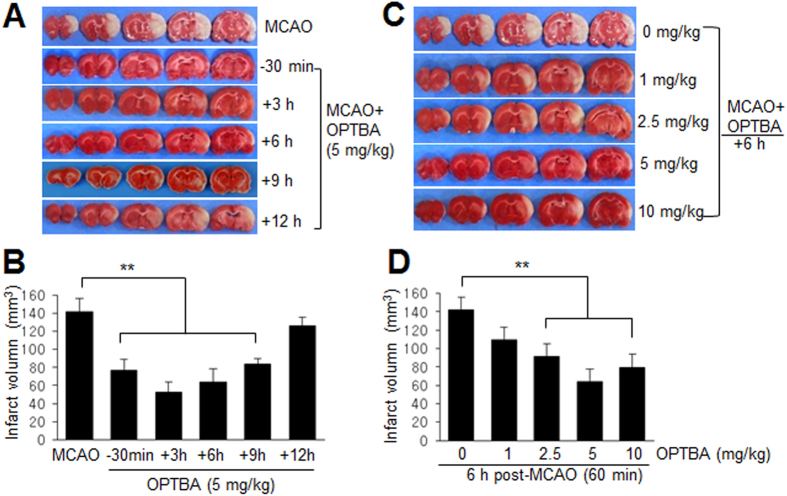
Neuroprotective effects of OPTBA in the postischemic brain. (**A**,**B**) OPTBA (5 mg/kg, i.v.) was administered at 30 min before or 3, 6, 9, or 12 h after MCAO. Mean infarct volumes were assessed at 2 days after MCAO by TTC staining. Representative images of infarctions in coronal brain sections are shown (**A**) and quantitative results are presented as means ± SEMs (n = 5–6) (**B**). (**C**,**D**) OPTBA (1, 2.5, 5, or 10 mg/kg, i.v.) was administered at 6 h after MCAO. Mean infarct volumes at 2 days after MCAO are presented as means ± SEMs (n = 5-6). **p < 0.01 differences between the indicated groups. MCAO, treatment-naive MCAO control rats; MCAO + OPTBA, OPTBA-administered MCAO rats.

To investigate the neuroprotective effect of OPTBA in the postischemic brain, OPTBA was administered at 5 mg/kg (i.v.) 30 min before or 3 or 6 h after MCAO (60 min) (Fig. 2A). Infarct volumes were found to be reduced to 55.4 ± 14.3% (n = 5, p < 0.01), 38.5 ± 11.4% (n = 5, p < 0.01), and 46.5 ± 15.3% (n = 6, p < 0.01), respectively, of that of treatment-naive MCAO controls (Fig. 2B). The administration of 5 mg/kg of OPTBA at 9 h after MCAO suppressed infarct volume to 59.0 ± 12.0% (n = 5, p < 0.01), indicating the neuroprotective effect of OPTBA in the postischemic brain had an extended therapeutic window (Fig. 2A,B). When OPTBA was administered i.v. at 1, 2.5, 5, or 10 mg/kg at 6 h after MCAO, infarct volumes were reduced to 78.7 ± 16.3% (n = 5, p > 0.05), 66.6 ± 21.2% (n = 6, p < 0.01), 46.5 ± 15.3% (n = 6, p < 0.01), and 57.1 ± 9.6% (n = 5, p < 0.01), respectively, of that of treatment-naive MCAO controls (Fig. 2C,D), which indicate that OPTBA administered at 5 mg/kg had the greatest protective effect.

### Neuroprotective potency of OPTBA was superior to that of the combined treatment of HTB and pyruvate

**Figure 3:**
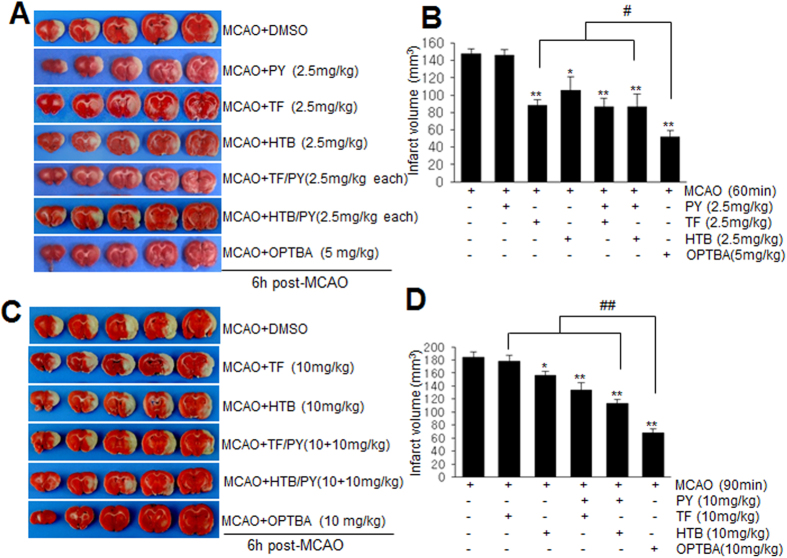
Comparison of the infarct suppression efficacies of OPTBA and of HTB (or triflusal) and/or pyruvate in the postischemic brain. (**A**,**B**) OPTBA (5 mg/kg), HTB (2.5 mg/kg), triflusal (TF, 2.5 mg/kg), pyruvate (PY, 2.5 mg/kg), HTB + pyruvate (2.5 mg/kg each), or triflusal + pyruvate (2.5 mg/kg each) were administered intravenously at 6 h after MCAO (60 min) and mean infarction volumes were assessed at 2 days after MCAO by TTC staining. (**C**,**D**) For 90 min MCAO, OPTBA (10 mg/kg), HTB (10 mg/kg), triflusal (TF, 10 mg/kg), HTB + pyruvate (10 mg/kg each), or triflusal + pyruvate (10 mg/kg each) were administered intravenously at 6 h after MCAO. Representative images of infarctions in coronal brain sections are presented (**A**,**C**) and quantitative results are presented as means ± SEMs (n = 5-8) (**B**,**D**). *p < 0.05, **p < 0.001 vs. MCAO group, ^#^*p* < 0.05 between indicated groups. MCAO, treatment-naive MCAO control rats; MCAO + PY, pyruvate-administered MCAO rats; MCAO + TF, triflusal-administered MCAO rats; MCAO + HTB, HTB-administered MCAO rats; MCAO + TF/PY, triflusal/pyruvate-co-administered MCAO rats; MCAO + HTB/PY, HTB/pyruvate-co-administered MCAO rats; MCAO + OPTBA, OPTBA-administered MCAO rats.

To compare the neuroprotective potencies of OPTBA and co-treatment with HTB and pyruvate, we administered 2.5 mg/kg of pyruvate and/or HTB (or triflusal (TF), i.v.) at 6 h after MCAO (Fig. 3A). Infarct volumes in the MCAO + TF/PY or MCAO + HTB/PY group were reduced to 59.2 ± 8.2% (n = 5, p < 0.01) and 60.2 ± 10.4% (n = 6, p < 0.01), respectively, of treatment-naive MCAO controls, which were similar to those in the MCAO + TF or MCAO + HTB group (Fig. 3A,B), indicating a lack of synergism in between pyruvate and HTB or between pyruvate and triflusal. However, OPTBA suppressed mean infarct volume to 35.6 ± 5.8% (n = 5, p < 0.01) of that of treatment-naive MCAO controls (Fig. 3A,B), indicating a significantly greater effect. It was notable here that neuroprotective effect of HTB was weaker than triflusal at 2.5 mg/kg (Fig. 3A,B), however, it was greater than triflusal at 5 mg/kg (Kim *et al*., submitted). Therefore, we investigated the neuroprotective effects of HTB, triflusal, and OPTBA at higher dose (10 mg/kg). For this, 90 min MCAO model was used, in which infarct volume was increased by 24.9 ± 9.6% compared to 60 min MCAO. Infarct volumes in MCAO + TF/PY (10 + 10 mg/kg) and MCAO + HTB/PY (10 + 10 mg/kg) groups were reduced to 69.6 ± 6.2% (n = 5) and 58.7 ± 3.6% (n = 5), respectively (Fig. 3C,D). Importantly, administration of 10 mg/kg of OPTBA reduced infarct volume to 35.4 ± 3.4% (n = 5, p < 0.01), which was significantly smaller not only than those of MCAO + TF or MCAO + HTB group but of MCAO + TF/PY or MCAO + HTB/PY group (Fig. 3C,D). These results further confirmed the neuroprotective potency of OPTBA in the postischemic brain was superior to the combined treatments with HTB/pyruvate or triflusal/pyruvate.

### OPTBA improved neurological deficits and motor impairment after MCAO

**Figure 4:**
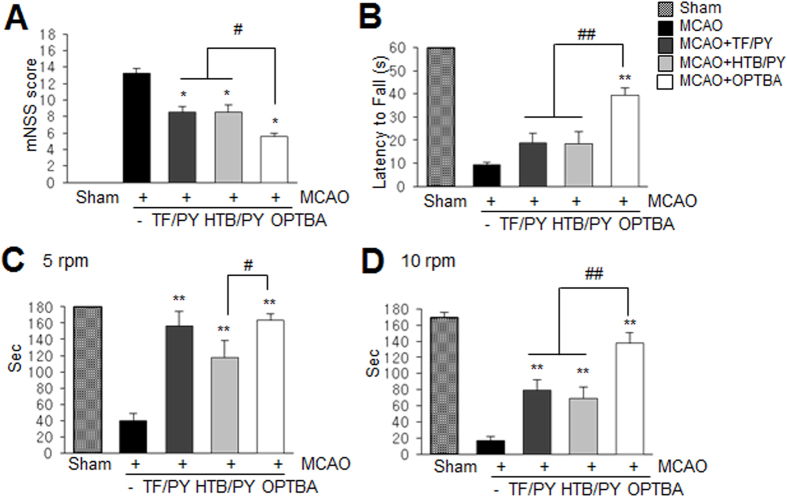
Preventions of neurological deficits and motor impairment. OPTBA (5 mg/kg, i.v.), HTB + pyruvate (2.5 mg/kg each), or triflusal + pyruvate (2.5 mg/kg each) was administered at 6 h after MCAO. (**A**) Neurological deficits were evaluated using mNSSs at 2 days after MCAO. Non-parametric Kruskal-Wallis H-test and Tukey’s test were performed and significance was set at p < 0.05. (**B**) Motor impairment was evaluated using a wire hanging test at 2 days after MCAO. (**C**,**D**) The rota-rod test was performed at 2 days after MCAO. Residence times on the spindle were recorded at spindle speeds of 5 and 10 rpm with a 1 h rest period after testing at 5 rpm. Sham, sham-operated rats; MCAO, treatment-naive MCAO control rats; MCAO + TF/PY, triflusal/pyruvate-co-administered MCAO rats; MCAO + HTB/PY, HTB/pyruvate-co-administered MCAO rats; MCAO + OPTBA, OPTBA-administered MCAO rats. Results are presented as means ± SEMs (n = 6–9). **p < 0.01 vs. MCAO group, ^#^*p* < 0.05, ^##^*p* < 0.01 between indicated groups.

Neurological deficits were evaluated using modified neurological severity scores (mNSSs) at 2 days after MCAO. mNSSs were significantly lower in MCAO + OPTBA (5 mg/kg) group (5.6 ± 0.4) (n = 9, p < 0.01) than in treatment-naive MCAO controls (13.3 ± 0.6) (n = 8) (Fig. 4A). Motor activities were assessed using a wire hanging test (Fig. 4B) and a rota rod test (Fig. 4C,D). In the wire hanging test, mean hanging time of the MCAO + OPTBA (5 mg/kg) group (39.4 ± 3.1 sec) (n = 9, p < 0.01) was significantly greater than that of MCAO + HTB/PY (2.5/2.5 mg/kg) group (18.5 ± 4.2 sec) (n = 6, p < 0.01) or MCAO + TF/PY (2.5/2.5 mg/kg) group (18.9 ± 3.9 sec) (n = 7, p < 0.01) (Fig. 4B). At a rota-rod speed of 5 rpm, difference in mean time stay on rota-rod was observed between the MCAO + HTB/PY (2.5/2.5 mg/kg) group (117.8 ± 13.8) (n = 6, p < 0.01) and MCAO + OPTBA (5 mg/kg) group (163.4 ± 7.7) (n = 7, p < 0.01) (Fig. 4C). Similarly, at 10 rpm, mean time was significantly greater in the MCAO + OPTBA (5 mg/kg) group (137.6 ± 13.1) (n = 7, p < 0.01) than in the MCAO + TF/PY (2.5 + 2.5 mg/kg) group (79.2 ± 12.8) (n = 5, p < 0.01) and in the MCAO + HTB/PY (2.5 + 2.5 mg/kg) group (68.8 ± 10.5) (n = 6, p < 0.01) (Fig. 4D). These results show that improvements in neurological deficits and motor impairment by OPTBA were superior to those achieved by co-administrating HTB and pyruvate or triflusal and pyruvate. Physiological parameters, including PaO_2_, PaCO_2_, pH, and blood glucose, were similar in OPTBA-treated and -untreated animals (Table 1).

### OPTBA suppressed inflammatory processes in postischemic brains

**Figure 5:**
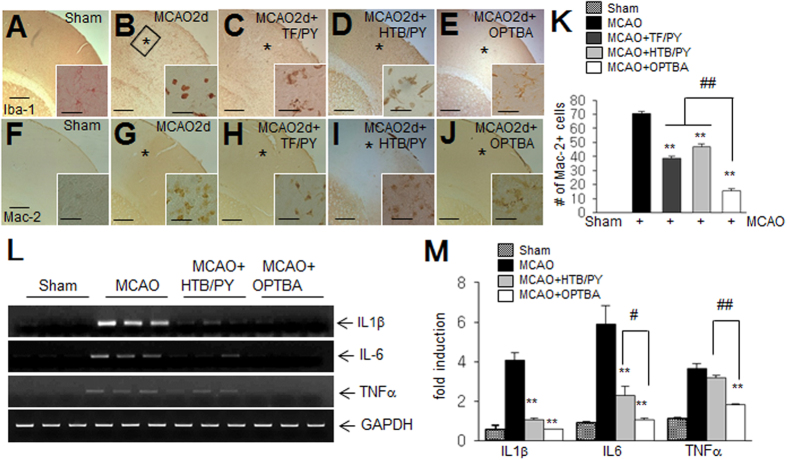
Suppression of inflammation by OPTBA in the postischemic brain. (**A**–**H**) Activated microglia was visualized by immunostaining with anti-Iba-1 (**A**–**E**) or anti-Mac-2 (**F**–**J**) antibodies in sham-operated (**A**,**F**), in MCAO control (**B**,**G**), in MCAO + TF/PY (**C**,**H**), in MCAO + HTB/PY (**D**,**I**), or in MCAO + OPTBA (**E**,**J**) rats at 2 days after MCAO. The insets are high magnification photographs of the indicated regions (*). Photographs are representative of three independent results obtained from three animals per each group. Scale bars in A-J represent 1 mm and the ones in inset of each photograph represent 100 μm. (K) Mac-2 positive cells in indicated regions (0.1 mm^2^ (0.32 × 0.32 mm), (*) were counted and results are presented as means ± SEMs (n = 12 from 3 animals). (**L**,**M**) The expressions of pro-inflammatory cytokines were examined at 1 day after MCAO. Samples for RT-PCR were prepared from indicated region (the black box in B) and changes in the RNA levels of IL-1β, IL-6, and TNFα are presented as means ± SEMs (n = 3). **p < 0.01 vs. MCAO group, ^#^*p* < 0.05, ^##^*p* < 0.01 between indicated groups. Sham, sham-operated rats; MCAO, treatment-naive MCAO control rats; MCAO + TF/PY, triflusal/pyruvate-co-administered MCAO rats; MCAO + HTB/PY, HTB/pyruvate-co-administered MCAO rats; MCAO + OPTBA, OPTBA-administered MCAO rats.

Because pyruvate and triflusal are known to exert anti-inflammatory effect^11^,^16^, we examined whether anti-inflammatory effect was responsible for the neuroprotective effect of OPTBA in the postischemic brain. First, we examined microglial activation using antibodies against Iba-1 (a marker of cells of myeloid origin)^17^ and Mac-2 (a marker of activated resident microglia)^18^. In sham animals, Iba-1 positive cells had a ramified morphology (Fig. 5A). At 2 days after-MCAO, Iba-1 positive cells were round and enlarged (indication of a phagocytic state) in treatment-naïve MCAO controls (Fig. 5B). However, most Iba-1 positive cells in the MCAO + OPTBA (5 mg/kg) group displayed a more ramified morphology and these ramified morphologies observed in OPTBA-treated animals were more evident than those in the MCAO + TF/PY (2.5 + 2.5 mg/kg) or MCAO + HTB/PY (2.5 + 2.5 mg/kg) group (Fig. 5C–E). In addition, numbers of Mac-2 positive cells in the MCAO + OPTBA (5 mg/kg) group (15.7 ± 1.2 cells in 0.1 mm^2^) (n = 12, p < 0.01) were significantly lower not only than that in the treatment-naïve MCAO controls (70.6 ± 1.5 cells per 0.1 mm^2^) (n = 12, p < 0.01) but those in MCAO + HTB/PY (2.5 + 2.5 mg/kg) group (46.8 ± 2.1 cells in 0.1 mm^2^) (n = 12, p < 0.01) or MCAO + TF/PY (2.5 + 2.5 mg/kg) group (38.8 ± 1.5 cells in 0.1 mm^2^) (n = 12, p < 0.01) (Fig. 5F–K). Moreover, the administration of OPTBA (5 mg/kg) at 6 h after MCAO suppressed the inductions of IL-1β, IL-6, and TNFα mRNA more effectively than HTB/PY (2.5 + 2.5 mg/kg) in the postischemic brain (Fig. 5L,M). These results show OPTBA had a robust anti-inflammatory effect in the postischemic brain.

### OPTBA suppressed microglia activation and pro-inflammatory cytokine induction by inhibiting IκBα degradation in cortical slice cultures

**Figure 6:**
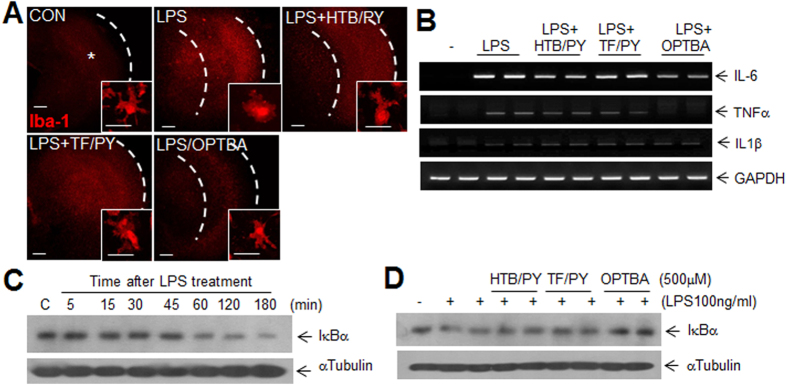
Anti-inflammatory effects of OPTBA in cortical slice culture. (**A**) LPS (100 ng/ml)-induced microglial activation was visualized by immunofluorescent staining with anti-Iba-1 antibody in the presence or absence of OPTBA (500 μM), HTB/PY (500 μM each), or TF/PY (500 μM each) in cortical slice cultures (n = 3). The insets are high magnification photographs of the indicated regions (*). Scale bars in (**A**) represent 250 μm and the ones in inset of each photograph represent 25 μm. (**B**) The expressions of pro-inflammatory cytokines after treatment with LPS (100 ng/ml) for 24 h in the presence or absence of OPTBA (500 μM)), HTB/PY (500 μM each), or TF/PY (500 μM each) were examined. (**C**) Degradation of IκB was examined by immunoblotting after treatment with LPS (100 ng/ml) for 0, 5, 15, 30, 45, 60, 120, and 180 min. (**D**) The effects of OPTBA (500 μM), HTB/PY (500 μM each), or TF/PY (500 μM each) on IκB degradation were investigated by immunoblotting after 3 h of LPS (100 ng/ml) treatment. α-Tubulin was used as a loading control and photographs shown are representative of three independent experiments.

To confirm the anti-inflammatory effect of OPTBA and investigate the underlying molecular mechanism, cortical slice cultures were treated with LPS (100 ng/ml) in the presence or absence of OPTBA (500 μM), HTB/PY (500 μM each), or TF/PY (500 μM each). Immunofluorescence staining showed Iba-1 immunoreactivity increased in LPS-treated slice cultures (Fig. 6A) and that microglia exhibited an activated morphology (an amoeboid shape) (insets in Fig. 6A). However, in OPTBA-treated cortical slice cultures, Iba-1 immunoreactivity was weaker (Fig. S2) and the amoeboid morphology was less evident not only than that in LPS-treated culture but than those in HTB/PY- or TF/PY co-treated cultures (Fig. 6A). In addition the inductions of IL-6, TNFα, and IL-1β observed in LPS-treated cortical slice cultures were also suppressed in OPTBA-co-treated cultures and importantly, wherein levels of these pro-inflammatory cytokines were significantly lower than those in HTB/PY- or TF/PY (500 μM)-co-treated cultures (Fig. 6B). Moreover, OPTBA (500 μM) suppressed the IκB degradation observed after 3 h of LPS treatment (100 ng/ml) more efficiently than HTB/PY- or TF/PY-co-treatment (Fig. 6C,D). Together, these results indicate OPTBA exerts its anti-inflammatory effect by inhibiting IκB degradation.

### OPTBA suppressed neuronal cell death in NMDA-treated hippocampal slice cultures

**Figure 7:**
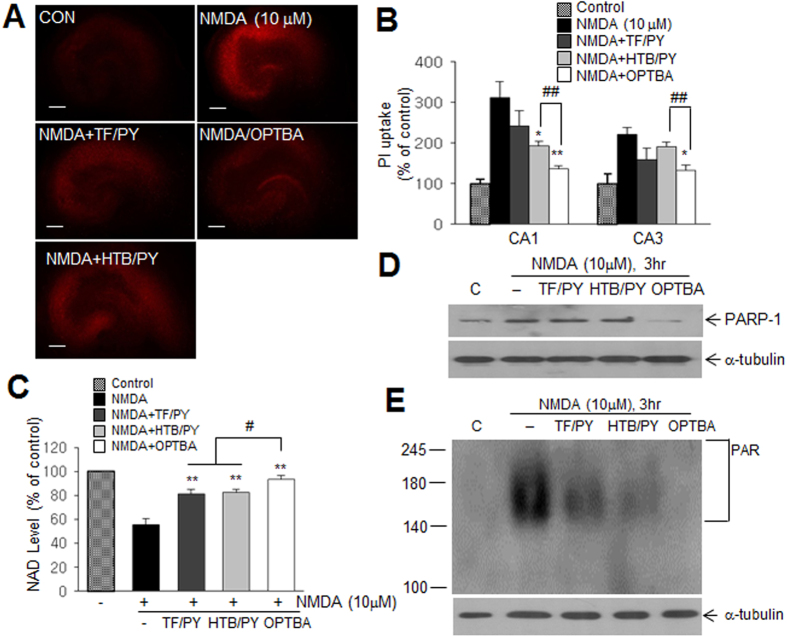
Anti-excitotoxic effects of OPTBA in hippocampal slice culture. (**A**) Cell deaths in hippocampal CA1 and CA3 regions in hippocampal slice cultures after 24 h of NMDA treatment (10 μM) in the presence or absence of OPTBA (250 μM), TF/PY (250 μM each), or HTB/PY (250 μM each) were visualized by propidium iodide (PI) staining. Scale bars in (**A**) represent 250 μm. (**B**) Fluorescence intensities measured using image J software in CA1 and CA3 are presented as means ± SEMs (n = 5). (C) Levels of NAD in hippocampal slice cultures were measured after 24 h of NMDA (10 μM) treatment in the presence or absence of OPTBA (250 μM), HTB/PY (250 μM each), or TF/PY (250 μM each). (**D**,**E**) PARP1 protein level (D) or PAR formation (**E**) was measured after 3 h of NMDA (10 μM) treatment in the presence or absence of OPTBA (250 μM), HTB/PY (250 μM each), or TF/PY (250 μM each). Results are presented as means ± SEMs (n = 5). *p < 0.05, **p < 0.01 vs. NMDA-treated group, ^#^*p* < 0.05, ^##^*p* < 0.01 between indicated groups.

Next, we investigated whether OPTBA also confers neuroprotective effect against excitotoxicity. In hippocampal slice cultures treated with NMDA (10 μM, 24 h), propidium iodide (PI) staining revealed significant increase of neuronal cell death, however, it was suppressed by OPTBA (250 μM) (Fig. S3) and the efficacy was higher than those of HTB/PY (250 μM each) or TF/PY co-treatment (250 μM each) (Fig. 7A,B). NMDA receptors regulate neuronal PARP 1 expression and activity, which causes cell death via depletions of NAD and ATP^19^. NAD depletion in NMDA-treated hippocampal slice cultures was suppressed by co-treating OPTBA (250 μM) and replenished NAD level in OPTBA (250 μM)-treated culture was higher than those in HTB/PY (250 μM each)- or TF/PY (250 μM each)-co-treated cultures (Fig. 7C). Furthermore, OPTBA suppressed PARP-1 protein induction far more effectively than TF/PY- or HTB/PY-co-treatment and PAR formation (Fig. S4) was also significantly suppressed by OPTBA (Fig. 7D,E). These results indicated that OPTBA exerted a marked neuroprotective effect against excitotoxic stress and it might also contribute to a robust neuroprotection observed in the postischemic brain.

## Discussion

In the present study, we showed OPTBA, a HTB-pyruvate ester, exerts a robust neuroprotective effect in the postischemic brain and that this was achieved by its anti-inflammatory and anti-excitotoxic effects probably accomplished directly by OPTBA and by its hydrolysis products, HTB and pyruvate. Among various cells involved in induction and aggravation of inflammation in the postischemic brain^20^, activated microglia probably play a key role by producing various neurotoxins, such as, nitric oxide, reactive oxygen species, and cytokines^21^,^22^,^23^,^24^. In the present study, OPTBA was found to suppress microglial activation more efficiently than the combined treatment of HTB and pyruvate in rat MCAO model (Fig. 5D,H) and in LPS-treated cortical slice cultures (Fig. 6A). HTB is a long-lasting active metabolite of triflusal and has been shown to inhibit COX-2 activity and the translocation of NF-κB, and thus, inhibit the de novo expressions of genes targeted by NF-κB^8^,^11^. Regarding the protective potency, it has been reported that overall neuroprotective effect of HTB in rat MCAO model is superior to triflusal and salicylic acid and that in particular, anti-inflammatory effects are also greater than them (Kim *et al*., Submitted). Since pyruvate is also known to block the infiltration of immune cells into the postischemic brain and inhibit LPS-induced microglial activation^16^, the anti-inflammatory potency of OPTBA could be contributed by the dual and complementary anti-inflammatory effects of HTB and pyruvate released from OPTBA after its hydrolysis.

In this regard, it is worthy of noting that HTB and pyruvate were released from OPTBA hydrolysis with a prolonged time window; the t_1/2_ was 38.6 min in blood and 7.2 and 2.4 h in brain parenchyma (Fig. 1). More importantly, plasma HTB level surged to the level higher than that of OPTBA 15 min after OPTBA injection, and the enhanced HTB level lasted for longer than 24 h both in blood and brain tissue (Fig. 1). These observations agree well with a previous report showing that HTB is stably detected in rat plasma (t_1/2_ of 21.5 h)^25^. We found that when OPTBA is administered intravenously as a single bolus, it can be hydrolyzed into HTB and pyruvate in blood and then enter the brain, and alternatively, it is possible for OPTBA to enter the brain parenchyma and then it is hydrolyzed. Considering that the high HTB level was rapidly achieved after OPTBA injection in blood (Fig. 1B) and that OPTBA accumulated almost immediately but its hydrolysis was slower in brain parenchyma (Fig. 1C), the neuroprotective effect of OPTBA is likely to be achieved through the rapid and sustained provision of HTB and pyruvate from the OPTBA hydrolysis and also by the prolonged action of HTB thanks to its remarkable stability (Fig. 1).

It has been previously reported pyruvate markedly reduced infarct formation in the postischemic brain^15^,^26^ and that it effectively replenished NAD levels in Zn^2+^-treated cortical neurons and scavenged hydrogen peroxide^13^,^27^. However, in previous studies, high doses of pyruvate (62.5–1000 mg/kg, i.p.) were required to suppress brain damage in the postischemic rat brain^15^,^16^,^28^ and millimolar concentrations of pyruvate were required to obtain neuroprotective effects in NMDA- or Zn^2+^-treated neuronal cells^13^,^29^,^30^. However, in the present study, OPTBA at 5 mg/kg reduced infarct volumes to 35.5 ± 12.3% versus MCAO controls when administered 6 h after MCAO (Figs 2 and 3), and micromolar concentration of OPTBA conferred neuroprotective effects in NMDA or LPS-treated slice cultures (Figs 6 and 7). In view of the fact that pyruvate is spontaneously converted to parapyruvate, an inhibitor of a key step in the tricarboxylic acid cycle^31^,^32^, the efficacy of OPTBA at lower dosages appears to add a distinct advantage.

In addition to its anti-inflammatory effect, OPTBA was found to exhibit superior anti-excitotoxic effects compared to that of combined treatment of HTB and pyruvate in NMDA-treated hippocampal slice cultures. It has been previously reported that exogenous pyruvate prevented neuronal degeneration by replenishing NAD in Zn^2+^-treated cortical neurons^13^ and ATP levels in NMDA-treated slice cultures^30^. In a separate study, we also found that both triflusal and HTB inhibited neuronal cell death in NMDA-treated cortical neurons and inhibition by HTB was greater than that by TF (Kim *et al*., Submitted). Hence, the higher efficacy of OPTBA in suppressing neuronal cell death in NMDA-treated hippocampal slice cultures was contributed by both pyruvate and HTB via suppressing PARP-1 expression and PAR formation (Fig. 7D,E). Thereby, we speculate that in NMDA-treated slice cultures, OPTBA supplies energy metabolites (ATP and NAD) in a sustained manner by stably producing pyruvate and HTB.

Since the brain damage caused by ischemic stroke is due to diverse pathophysiological events^33^, therapeutic strategies of choice are multimodal or combinatorial drug treatment. In the present study, we reported that OPTBA confers a robust neuroprotective effect in the postischemic brain, which was afforded by anti-inflammatory and anti-excitotoxic effects, and that the prolonged provisions of pyruvate and HTB by OPTBA hydrolysis enhance these effects.

## Materials and Methods

### Synthesis of 2-((2-Oxopropanoyl)oxy)-4-(trifluoromethyl)benzoic acid


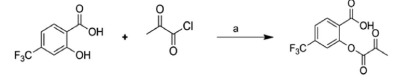


Pyruvoyl chloride (3.12 g, 29.3 mmol) was added to a solution of 2-hydroxy-4-(trifluoromethyl)benzoic acid (2.01 g, 9.75 mmol) and K_2_CO_3_ (4.04 g, 29.3 mmol) in acetone (150 mL) at 0 °C. The reaction mixture was stirred at room temperature for 4 h and quenched with 1 N HCl solution. The solution was extracted with ethyl acetate and the organic layer was washed with water and brine, dried over Na_2_SO_4_, and evaporated *in vacuo*. The crude residue was purified by column chromatography to give the title compound as a white solid (1.85 g, 68.8% yield). MP 275 °C; ^1^H NMR (CDCl_3_ + DMSO-*d*_*6*_) δ 1.97 (s, 3H), 7.32 (s, 1H), 7.35-7.40 (d, *J* = *8.0* *Hz*, 1H), 8.00-8.08 (d, *J* = *8.0* *Hz*, 1H); ^13^C NMR (CDCl_3_ + DMSO-*d*_*6*_) δ 23.34, 102.26, 114.26 (*J*_*C−F*_ = 3.8 Hz), 116.60, 119.62 (*J*_*C−F*_ = 3.8 Hz), 121.18 (*J*_*C−F*_ = 270.7 Hz), 130.36, 137.06 (*J*_*C−F*_ = 33.3 Hz), 155.88, 159.05, 168.21; MS [M-H]^−^ : 275.05.

### OPTBA, triflusal, HTB, or pyruvate injection to MCAO-operated rats

Sodium pyruvate, HTB, or triflusal (2.5 mg/kg each) was dissolved in 70% DMSO (50 μl) and administered intravenously at 6 h after MCAO. OPTBA (1, 2.5, 5, or 10 mg/kg) was administered intravenously in 50 μl of 70% DMSO at indicated time points. Animals were randomly divided into 10 groups, as follows: a Normal group (n = 10), treatment-naïve controls; DMSO (50 μl of 70% DMSO) + OPTBA group (n = 46), OPTBA-administered rats; a Sham group (n = 14), animals underwent surgery but were not subjected to MCAO; a MCAO group, treatment-naive MCAO controls (n = 41, 50 μl of DMSO (70%)-treated); a MCAO + PY group, pyruvate-administered MCAO rats (n = 6); a MCAO + TF group (n = 9), triflusal-administered MCAO rats; a MCAO + HTB group (n = 10), HTB-administered MCAO rats; a MCAO + TF/PY group (n = 25), triflusal/pyruvate-co-administered MCAO rats; a MCAO + HTB/PY group (n = 25), HTB/pyruvate-co-administered MCAO rats; and a MCAO + OPTBA group (n = 67), OPTBA-administered MCAO rats. No animal died during surgery, but overall mortality after surgery was 4.5% (12/265; 6, MCAO; 1, MCAO + TF; 2, MCAO + HTB; 1, MCAO + HTB/PY; 2, MCAO + OPTBA).

### Surgical procedures for MCA occlusion

All procedures concerning animals were carried out in strict accordance with the Guide for the Care and Use of Laboratory Animals published by the National Institute of Health (2010) and complied with Animal Research: Reporting of *In Vivo* Experiments (ARRIVE) guidelines (http://www.nc3rs.org/ARRIVE). The animal protocol used in this study was reviewed and approved by the INHA University-Institutional Animal Care and Use Committee (INHA-IACUC) with respect to ethicality (Approval Number INHA-141124-337-2). Nine week-old male Sprague-Dawley (SD) rats weighing 230–250 g were purchased from Orient Bio Inc (Gyeonggi, South Korea), housed under diurnal lighting conditions and allowed food and tap water ad libitum for 1 week. MCAO was carried out as previously described^6^. In brief, male Sprague-Dawley rats (250–300 g) were anesthetized with 5% isoflurane in a 30% oxygen/70% nitrous oxide mixture, anesthesia was maintained during procedures using 0.5% isoflurane in the same gas mixture. Animals were randomly allocated to the 10 treatment groups described in the previous section. MCA occlusion was performed for 60 min using a nylon suture (4-0; AILEE, Busan, South Korea) and was followed by reperfusion. During the procedure, the left femoral artery was cannulated to obtain a blood sample, which was analyzed for pH, PaO_2_, PaCO_2_, and blood glucose concentration (I-STAT; Sensor Devices, Waukesha, WI). Laser Doppler flowmetry (Periflux System 5000; Perimed, Jarfalla, Sweden) was used to monitor regional cerebral blood flow (CBF) and relative CBF during the experiment. Operated rats which did not show >70% reduction in CBF during MCAO were excluded from the experimental groups. A thermoregulated heating pad and a heating lamp were used to maintain a rectal temperature of 37.0 ± 0.5 °C during procedures. Investigators blinded to the experimental groups performed the behavioral analysis and the data analysis. Animals in the sham group were operated in an identical manner but the MCA was not occluded.

### Sample Preparation for LC/ESI-MS

OPTBA (5 mg/kg) was injected intravenously into treatment-naive animals. Plasma (0.25, 0.5, 1, 2, 4, 12 and 24 h) and brain tissue (cerebral cortex and striatum) samples (0.5, 1, 3, 6, 12, and 24 h) were collected after injection. Preparations and analysis of plasm and brain tissue (cerebral cortex and striatum) samples were carried out as described previously^6^. The pharmacokinetic characteristics of OPTBA were assessed by a noncompartmental method using Phoenix WinNonlin 6.4 (Certara, Princeton, NJ).

### Infarct volume assessment

Rats were decapitated at 2 days post-operation and whole brains were dissected coronally into 2-mm brain slices using a metallic brain matrix (RBM-40000, ASI, Springville, UT). Slices were immediately incubated in saline containing 2, 3, 5-triphenyl tetrazolium chloride (TTC, 2%) at 37 °C for 15 min and then in 4% paraformaldehyde. Areas of infarcted tissue were measured using the Scion Image program (Scion Image program, Frederick, MD). To adjust for edema and shrinkage, areas of ischemic lesions were calculated using (contralateral hemisphere volume x measured injury volume/ipsilateral hemisphere volume) and infarct volumes were quantified (in mm^3^) by multiplying summed infarct areas of sections by section thickness.

### Evaluation of modified neurological severity scores

Neurological deficits were evaluated using modified Neurological Severity Scores (mNSS) at 2 days after MCAO. The mNSS system consists of motor, sensory, balance, and reflex tests, all of which are graded using a scale of 0 to 18 (normal: 0, maximal deficit: 18)^34^.

### Wire hanging test

The wire hanging test procedure has been previously described^35^, and was used to measure forelimb strength and grasping ability at 2 days after MCAO. Briefly, a rat was suspended by its forelimbs on a horizontal steel wire (50 cm long, 2 mm diameter And after grasping the time to falling off was measured using a stopwatch up to a cutoff time of 60 s.

### Rota-rod test

One day before surgery, rats were trained on a rota-rod unit (Daejon Instruments, Seoul, Korea) at a constant 3 rpm until they were capable of remaining on the rotating spindle for 180 s. At 2 days after surgery, residence times on the spindle were recorded at spindle speeds of 5 and 10 rpm with a 1 h rest period after testing at 5 rpm.

### Immunohistochemistry

The animals were sacrificed at 2 days (n = 3 per group) after MCAO and brains were fixed using 4% paraformaldehyde (PFA) by transcardiac perfusion and post-fixed in the same solution overnight at 4 °C. Brain sections (40 μm) were prepared using a vibratome, and then immunologically stained using a previously described floating method^6^. Primary antibodies were diluted as follows; 1:300 for anti-ionized calcium binding adaptor molecule-1 (Iba-1) (Wako Pure Chemicals, Osaka, Japan) and 1:250 for anti-Mac2 (Abcam, Cambridge, UK). The images shown are representative of the results obtained from three animals for each group.

### RNA preparation and RT-PCR

Total RNA was prepared using TRIzol reagent (Gibco BRL, Gaithersburg, MD), and 1000 ng aliquots of RNA samples were used for cDNA synthesis, which was conducted using a RT-PCR kit (Roche, Mannheim, Germany). The sequences of the rat interleukin-1β (IL-1β), TNF-α, IL-6, and GAPDH primers used were described previously^36^.

### Organotypic hippocampal and cortical slice cultures

Rats were sacrificed at postnatal days 3 (cortex) or 7 (hippocampus). Brains were aseptically removed and cortices or hippocampi were dissected from hemispheres and cut into 350 μm slices using a McIlwain Tissue Chopper (The Mickle Laboratory Engineering Co., Surrey, UK) and subsequent procedures for organotypic Slice cultures were conducted as described previously^37^. The animal protocol used was also reviewed and approved by the INHA University Institutional Animal Care NA Use Committee (Approval Number INHA 140522-297-3). Hippocampal and cortical slice cultures were treated with TF/PY, HTB/PY, or OPTBA at 250 μM or 500 μM each, respectively.

### Propidium iodide (PI) staining

Neuronal cell death in OHSCs was determined by propidium iodide (PI) staining. Briefly, OHSCs were treated with NMDA (10 μM) for 24 h, PI (1 μg/mL) was then added and incubation continued for 30 min. OHSCs were then fixed in 4% paraformaldehyde (PFA) for 15 min and fluorescence was visualized under a Zeiss fluorescence microscopy (Axio Observer, Oberkochen, Germany). PI fluorescence intensities were measured in the hippocampal CA1 and CA3 regions using Image J software (National Institutes of Health, Bethesda, ML) and presented as fold increases versus NMDA non-treated control.

### NAD Level Determination

NAD concentrations were determined by using a cyclic enzymatic assay^38^.

### Immunoblotting

Slice cultures were washed twice with cold PBS and lysed in RIPA buffer containing 50 mM Tris-HCl (pH7.4), 1% NP-40, 0.25% sodium-deoxycholate, 150 mM NaCl, and complete Mini protease inhibitor cocktail tablets (Roche, Mannheim, Germany). Lysates were centrifuged for 15 min at 17,500 g at 4 °C and supernatants were loaded into 6–10% SDS PAGE gels. Primary antibodies for anti-IκB (Santa Cruz Biotechnology, Santa Cruz, CA), anti-PARP-1 (Santa Cruz Biotechnology, Santa Cruz, CA) and anti-PAR (Trevigen, Gaithersburg, MD) were diluted in 1:2000. Primary antibodies were detected using a chemiluminescence kit (Merck Millipore, Darmstadt, Germany) using horseradish peroxidase-conjugated secondary antibody (1:4000; Merck Millipore, Darmstadt, Germany).

### Immunofluorescent staining of cortical slice cultures

Cortical slices were fixed with 4% paraformaldehyde (PFA) and incubated in 1% Triton X-100 in phosphate-buffered saline (PBS) at for 24 h. Cortical slices were blocked in 20% bovine serum albumin (BSA) containing 0.1% TritonX-100 in PBS for 1 h at room temperature. Antibody for anti-Iba-1 (Wako Pure Chemicals, Osaka, Japan) was diluted at 1:200 in 1% normal goat serum containing 0.1% TritonX-100 in PBS and Cortical slices were treated with this antibody for 24 h. Cortical slices were then washed with PBS containing 0.1% Triton X-100, and incubated with Rhodamine-labeled anti-rabbit IgG (Jackson ImmunoRes, West Grove, PA) (1:300) for 3 h at room temperature. Fluorescence was visualized under a Zeiss microscopy (Axio Observer, Oberkochen, Germany).

### Statistical analysis

Statistical analysis was performed by analysis of variance (ANOVA) followed by the Newman-Keuls test. For a non-parametric statistics test, we performed non-parametric Kruskal-Wallis H-test and Tukey’s test on SPSS package 18. Results are presented as means ± SEMs, and statistical difference was accepted at the 5% level.

## Additional Information

**How to cite this article**: Kim, S.-W. *et al*. Robust neuroprotective effects of 2-((2-oxopropanoyl)oxy)-4-(trifluoromethyl)benzoic acid (OPTBA), a HTB/pyruvate ester, in the postischemic rat brain. *Sci. Rep*. **6**, 31843; doi: 10.1038/srep31843 (2016).

## Supplementary Material

Supplementary Information

## Figures and Tables

**Table 1:** Physiological parameters Values are means SD (n = 5).

	Vehicle-treated group (n = 5)		OPTBA-treated group (n = 5)	
	Base	During ischemia	Base	During ischemia
Rectal Temperature (°C)	37.3 ± 0.17	37.3 ± 0.16	37.3 ± 0.21	37.3 ± 0.21
pH	7.5 ± 0.03	7.52 ± 0.02	7.49 ± 0.02	7.48 ± 0.02
PO_2_ mmH	172.4 ± 4.5	170.4 ± 3.3	167.4 ± 3.0	170.4 ± 4.3
PCO_2_ mmHg	35.6 ± 1.1	33.6 ± 0.7	35.0 ± 0.8	33.5 ± 2.3
Glucose, mg/dL	113 ± 3.1	116.2 ± 3.0	115.8 ± 3.8	113.6 ± 3.5

OPTBA (5 mg/kg) or vehicle (DMSO) was administered i.v. 30 min before MCAO. One way analysis of variance revealed no significant intergroup difference for any variance.
