# Supplementary material for: Robust neuroprotective effects of 2-((2-oxopropanoyl)oxy)-4-(trifluoromethyl)benzoic acid (OPTBA), a HTB/pyruvate ester, in the postischemic rat brain
Source: Sci Rep. 2016 Aug 22;6:31843. doi: 10.1038/srep31843 (PMC4992820; doi:10.1038/srep31843)
Supplement: Supplementary Information [file srep31843-s1.pdf]

Supplementary information

Robust neuroprotective effects of 2-((2-oxopropanoyl)oxy)-4-(trifluoromethyl)benzoic acid (OPTBA), a HTB/pyruvate ester, in the postischemic rat brain

Seung-Woo Kim[1,3], Hye-Kyung Lee[2,3], Il-Doo Kim[2,3], Hahnbie Lee[2,3], Lidan Luo[2,3], Ju-Young Park[4], Sung-Hwa Yoon[4*], Ja-Kyeong Lee[2,3*]

[1]Department of Biomedical Sciences, Inha University School of Medicine, [2]Department of Anatomy, Inha University School of Medicine, [3]Medical Research Center, Inha University School of Medicine, Inchon, Korea; [4]Department of Molecular Science and Technology, Ajou University, Suwon, Republic of Korea

[*] They are equally contributed
[*] Corresponding author: **jklee@inha.ac.kr**

Fig. S1

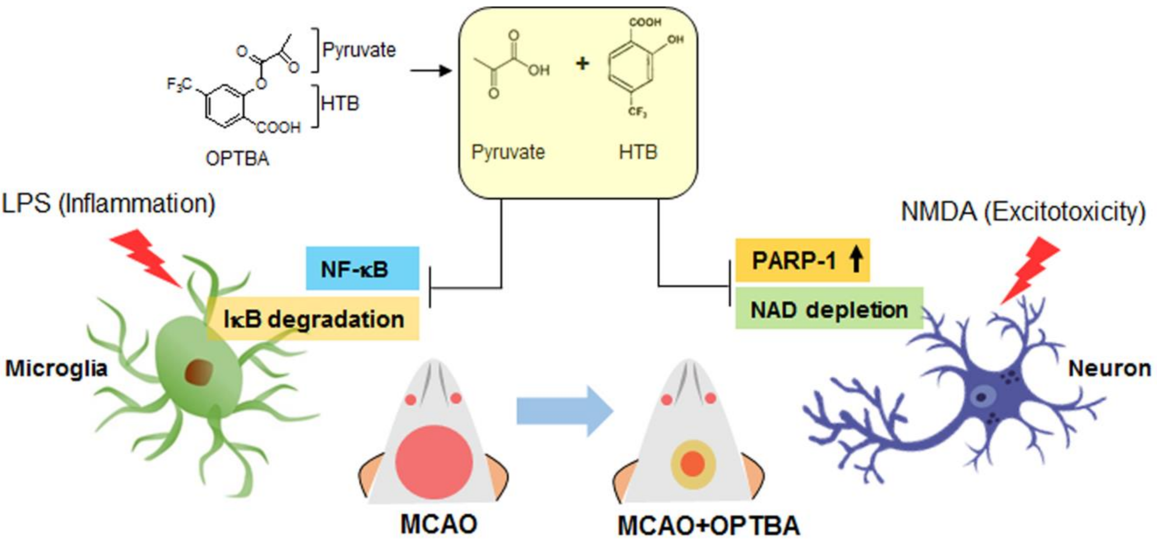

Pyruvate
HTB
OPTBA
Pyruvate
HTB
LPS (Inflammation)
NMDA (Excitotoxicity)
NF-κB
IκB degradation
PARP-1
NAD depletion
Microglia
Neuron
MCAO
MCAO+OPTBA

Fig. S2

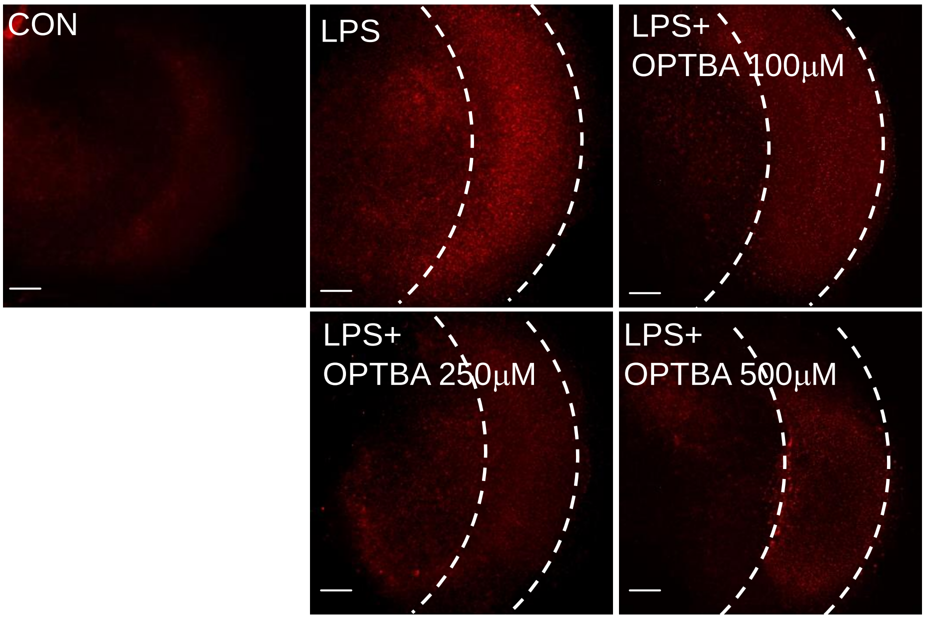

Fig. S3

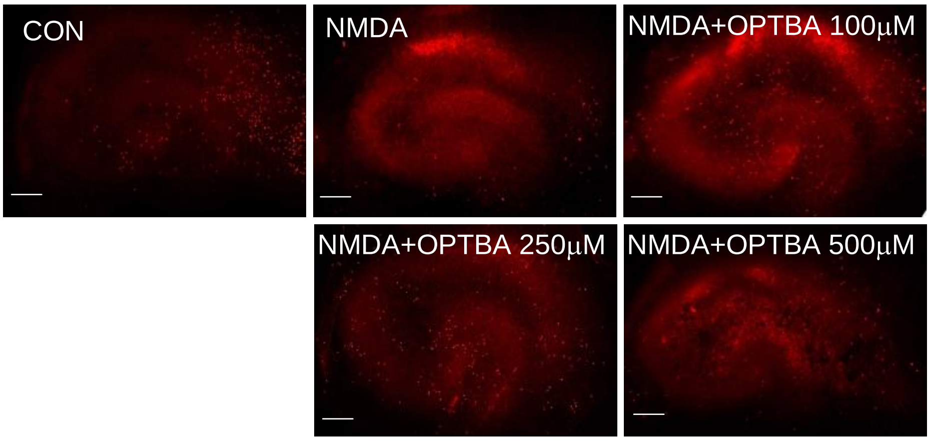

Fig. S4

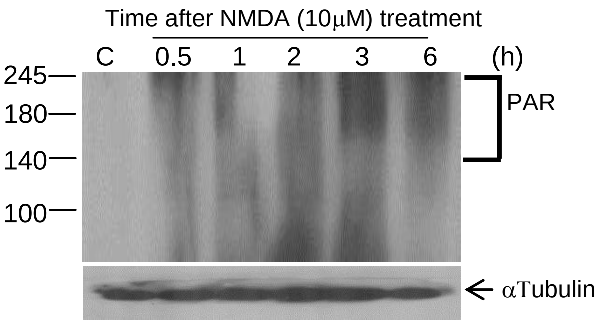
Time after NMDA (10μM) treatment
C
0.5
1
2
3
6
(h)
245
180
140
100
PAR
αTubulin

Figure S1. Mechanism of multi-modal neuroprotectant, OPTBA

Schematic diagram shows the neuroprotective effects of OPTBA via anti-inflammation and anti-excitotoxicity.

Figure S2. Dose dependent suppression of OPTBA on LPS-induced Iba-1 expression in cortical slice culture

LPS (100 ng/ml)-induced microglial activation was visualized by immunofluorescent staining with anti-Iba-1 antibody in the presence or absence of OPTBA (100, 250, and 500 μM). Scale bar, 250 μm.

Figure S3. Dose dependent suppression of OPTBA on NMDA-induced neuronal cell death in hippocampal slice culture

Cell death in hippocampal CA1 and CA3 regions in the presence or absence of OPTBA (100, 250, and 500 μM) was visualized by propidium iodide (PI) staining after treatment with NMDA (10 μM) for 24 h. Scale bar, 250 μm.

Figure S4. Activation of PARP-1 in hippocampal slice culture

PARP-1 activity was measured after 0.5, 1, 2, 3, or 6 h of NMDA (10 μM) treatment using anti-PAR antibody. α-tubulin was used as a loading control.
